# The Role of Quetiapine in Treating Delirium in Critical Care Settings: A Narrative Review

**DOI:** 10.3390/jcm14082798

**Published:** 2025-04-18

**Authors:** Alessandro Menozzi, Miriam Gotti, Elena Alessandra Mantovani, Andrea Galimberti, Michele Umbrello, Giovanni Mistraletti, Giovanni Sabbatini, Angelo Pezzi, Paolo Formenti

**Affiliations:** 1 School of Medicine and Surgery, University of Milano-Bicocca, 20126 Milano, Italy; a.menozzi2@campus.unimib.it; 2 Struttura Complessa Anestesia, Rianimazione e Terapia Intensiva, ASST Nord Milano, Ospedale Bassini, 20097 Cinisello Balsamo, Italy; miram.gotti@asst-nordmilano.it (M.G.); elena.mantovani@asst-nordmilano.it (E.A.M.); andrea.galimberti@asst-nordmilano.it (A.G.); giovanni.sabbatini@asst-nordmilano.it (G.S.); angelo.pezzi@asst-nordmilano.it (A.P.); 3 Department of Intensive Care, New Hospital of Legnano: Ospedale Nuovo di Legnano, 20025 Legnano, Italy; michele.umbrello@fastwevnet.it (M.U.); giovanni.mistraletti@unimi.it (G.M.)

**Keywords:** delirium, quetiapine, ICU, antipsychotics in ICU

## Abstract

Delirium is a frequent complication in critically ill patients, often leading to worse clinical outcomes, prolonged ICU stays, and an increased healthcare burden. Its identification has become more consistent with the adoption of validated diagnostic tools, allowing clinicians to recognize and address this condition more effectively. Although delirium can arise from direct neurological dysfunction, it is frequently a consequence of systemic conditions such as sepsis or organ failure. Therefore, a comprehensive evaluation of underlying causes is essential before initiating pharmacological treatment. Among the pharmacological options, quetiapine has gained attention for its use in ICU patients with delirium. Compared to first-generation antipsychotics, it is often preferred due to its sedative effects and more favorable safety. However, current clinical guidelines remain inconclusive regarding its routine use, as evidence supporting its efficacy is limited. One of the main challenges is the heterogeneity of patient populations included in randomized trials, making it difficult to determine whether specific subgroups may benefit more from treatment. This narrative review explores the pharmacological properties of quetiapine, its potential role in managing ICU delirium, and the current state of evidence regarding its safety and effectiveness.

## 1. Introduction

Delirium is a common condition in intensive care unit (ICU) patients, posing significant challenges to patient care and its outcome. This condition often leads to extended hospital stays, prolonged dependence on mechanical ventilation, and subsequently higher healthcare costs [1,2,3,4]. More critically, it contributes to higher rates of morbidity and mortality among this vulnerable population [5].

Over the years, the diagnosis of delirium in the ICU has become more standardized and reliable, thanks to the implementation of generally accepted assessment tools such as the confusion assessment method for ICU (CAM ICU) [4] or and the Intensive Care Delirium Screening Checklist (ICDSC) [6]. The Richmond Agitation-Sedation Scale (RASS) can be utilized to evaluate a patient’s agitation or sedation levels, serving as an initial assessment tool rather than a diagnostic instrument for delirium [3]. Once an appropriate level of consciousness is established using the RASS, clinicians can employ validated tools such as CAM-ICU or the ICDSC to accurately diagnose delirium. This two-step approach ensures that patients are appropriately stratified and assessed, leading to the more accurate identification and management of delirium in critical care settings. Other promising tools are the PRE-DELIRIC and e-PRE-DELIRIC models for predicting delirium risk in intensive care patients, consisting of several risk factors and showing a high predictive value for the diagnosis of delirium [7,8,9]. Based on this assessment, appropriate therapeutic interventions can be evaluated and eventually applied. Nevertheless, it is crucial to acknowledge that delirium may, in certain cases, represent an epiphenomenon of underlying conditions, such as sepsis or other organ dysfunctions, which are not necessarily linked directly to a primary neurological disorder [10,11]. Infections can lead to systemic inflammation and altered mental status. Systemic inflammation triggers the release of proinflammatory cytokines which can penetrate the blood–brain barrier and activate microglial cells in the brain [12]. Once activated, microglia produce more inflammatory cytokines and reactive oxygen species, exacerbating inflammation in the brain and potentially impairing normal brain function, which may lead to delirium [13]. Metabolic imbalances, such as electrolyte disturbances due to sodium and calcium, can directly affect neuronal function, modifying excitability and neurotransmission [14]. Certain medications, particularly those with anticholinergic properties or sedative effects, may exacerbate cognitive impairment. Sleep deprivation disrupts circadian rhythms and cognitive processes, further increasing the risk of delirium [15]. Additionally, immobility can lead to deconditioning and cognitive decline, while environmental factors, such as excessive noise or inadequate lighting, can contribute to disorientation [16]. Hence, before considering pharmacological therapy, which carries its own costs and potential side effects, it is essential to carefully explore and address any possible underlying causes of delirium. Although the current guidelines do not provide a definitive stance on their use, a wide array of antipsychotics drugs have been studied for their efficacy in treating ICU delirium, leading to diverse prescribing practices across different medical institutions. Among these medications, quetiapine stands out for its off-label use in managing ICU delirium [17]. Its popularity in this context is largely attributed to its sedative properties and a generally more favorable side effect profile compared to older, first-generation antipsychotics [18,19,20]. Nevertheless, current guidelines provide a conditional recommendation regarding the use of antipsychotics for treating delirium in adult ICU patients, indicating that there is insufficient evidence to support or refute its routine use [21]. This is due to methodological approaches, since most RCTs include a mix of patients with either hypoactive or hyperactive delirium, making it difficult to determine whether a specific subgroup would benefit more from antipsychotic treatment. Consequently, this remains an open issue, leaving the final judgment to clinical experience and the specific context. Therefore, we considered it appropriate to develop a narrative review that integrates scientific literature with clinical experience. For this reason, in this narrative review, we aim to provide a concise overview of the mechanisms of action of quetiapine, its potential benefits and adverse effects, and its most recent indications in the ICU setting. We also support the available evidence with a practical algorithm which, in the absence of stronger data, could help to guide clinicians in making the most appropriate decisions.

## 2. Literature Selection

In this narrative review, we selected the relevant literature through a structured search of six major databases: PubMed (1996–present), Embase (1974–present), Scopus (2004–present), SpringerLink (1950–present), Ovid Emcare (1995–present), and Google Scholar (2004–present). We included studies that provided original data or comprehensive analyses on quetiapine and delirium, focusing primarily on randomized controlled trials, observational studies, and meta-analyses. The search strategy involved using keywords such as “quetiapine”, “delirium”, “quetiapine and delirium”, and “quetiapine in ICU” to identify relevant studies. Two authors (AM and PF) were responsible for retrieving and reviewing the full texts of articles that met the search criteria. They carefully examined titles and abstracts to determine relevance and obtained full-text versions for detailed evaluation. The quality of the selected articles was assessed by evaluating their methodology, sample size, study design, and relevance to the topic of quetiapine use in delirium. Given the nature of a narrative review, we did not perform a formal quality assessment of the included studies, and selection was based on relevance and scientific merit. While this approach allows for a broader discussion of the topic, it also presents limitations compared to systematic reviews, as it may be subject to selection bias and does not provide a quantitative synthesis of the evidence. The included studies were then grouped thematically and critically analyzed to synthesize the key findings, identify knowledge gaps, and provide a comprehensive overview of the current understanding and clinical implications of quetiapine in the management of delirium.

## 3. Definition and Characteristics of Delirium

In this review, we refer to delirium as defined by the Diagnostic and Statistical Manual of Mental Disorders (DSM-5) and diagnosed in the ICU setting using validated tools such as the Confusion Assessment Method for the ICU (CAM-ICU) and the Intensive Care Delirium Screening Checklist (ICDSC). Delirium is defined as an acute syndrome which includes multiple features such as disturbance in attention (i.e., reduced ability to direct, focus, sustain, and shift attention) and awareness (reduced orientation to the environment) [22]. It is essential to emphasize that delirium implies an acute change from baseline attention and awareness, often fluctuating in severity throughout the day [23]. Additionally, it may manifest as disturbances in cognition, including memory impairment, disorientation, language difficulties, visuospatial deficits, or perceptual alterations [24].

Patients with delirium often experience disorientation and difficulty with complex tasks. Due to its fluctuating nature, they may have periods of lucidity interrupted by episodes of confusion [25]. Importantly, delirium differs from coma, as even in its hypoactive form, it does not involve a severely reduced level of arousal [26]. It is now widely recognized that the etiology of delirium is likely multifactorial, with factors such as older age and cognitive impairment contributing to its development [27]. Other predictive factors include the presence of multiple conditions associated with coma, the use of sedative medications (e.g., benzodiazepines), analgesics (e.g., opioids and ketamine), increased severity of illness, emergency type of admission as opposed to planned, admission diagnoses (such as neurological, neurosurgical, and trauma cases), presence of infection, dehydration, malnutrition, renal failure, and the use of hemofiltration [28,29,30].

The pathophysiological mechanisms underlying delirium may vary depending on the cause and likely involve multiple pathways including the GABAergic and cholinergic neurotransmitter systems. This is supported by the increased risk associated with the use of GABA agonists and anticholinergic drugs [31]. Medication side effects account for up to 39% of delirium cases; therefore, great care should be taken in selecting the most appropriate drug [32]. The primary neurotransmitter disorders associated with delirium include a deficit in acetylcholine and/or melatonin, an excess of dopamine, norepinephrine, and/or glutamate, and variable changes in serotonin, histamine, and γ-aminobutyric acid (GABA) activity, which can fluctuate based on the presentation and underlying cause of delirium [33].

It is improbable that any single theory can comprehensively account for the etiology or phenomenological manifestations of delirium [34]. Instead, it is more likely that two or more of these factors, if not all, interact to cause the biochemical disturbances that lead to the complex cognitive and behavioral changes involved in delirium [35].

## 4. Clinical Features of Delirium in ICU

Delirium can manifest in different forms, which are generally categorized based on the predominant behavioral aspects in a hyperactive and a hypoactive form. Hyperactive delirium is characterized by agitation and restlessness, making it relatively easy to recognize due to the evident behavior changes [36]. In contrast, hypoactive delirium is often slighter, with patients appearing withdrawn, mute, and drowsy. This subdued presentation can sometimes lead to it being overlooked, as it may not initially appear problematic [37]. Mixed delirium includes features of both hyperactive and hypoactive delirium, with patients fluctuating between the two states [38]. Additionally, heightened sympathetic activity can lead to physical manifestations, including hypertension and tachycardia [24].

In ICU, delirium frequently emerges as an epiphenomenon of underlying conditions rather than as a direct result of primary neurological disorders [39]. Sepsis, for instance, is a common precipitating factor, where systemic infection triggers a cascade of inflammatory responses leading to delirious states [40]. Similarly, cardiopulmonary abnormalities, such as acute respiratory failure or cardiac dysfunction, can significantly disrupt cerebral homeostasis, manifesting as delirium [41].

Finally, it is essential to consider that similar symptoms may also arise from physical and environmental factors specific to ICU care, such as mechanical ventilation, communication barriers due to orotracheal intubation, poor sleep hygiene, inadequate pain management, and prolonged immobilization [42,43].

## 5. Applications, Dosages, and Key Consideration in ICU Management

Given the factors discussed, quetiapine has become commonly used in ICU settings to manage delirium [44]. It is rapidly absorbed, being administered enterally via nasogastric tube and reaching peak plasma levels within an hour, with a primary half-life of seven hours. The extended duration of action, aided by its active metabolite N-desalkyl quetiapine (half-life of around twelve hours), helps to ensure continued symptom management [45]. Metabolized in the liver by the enzyme CYP3A4, quetiapine’s effectiveness and appropriate dosage depend significantly on liver function [46]. At lower doses, quetiapine primarily acts as a sedative by blocking histamine H1 and muscarinic receptors, aiding sleep and reducing anxiety with minimal risk of worsening delirium [47]. It also partially activates 5-HT1A receptors, enhancing its sedative effect. At higher doses, its stronger impact on dopamine D2 and serotonin 5-HT2A receptors aligns it with other atypical antipsychotics, making it effective for more severe agitation and delirium [48].

Table 1 summarizes the studies specifically focused on critically ill ICU patients that address key aspects of delirium assessment, pharmacological management, and related clinical outcomes Few studies have explored the efficacy of quetiapine as a sedative alone in mechanically ventilated patients. Ohman et al. [49] aimed to evaluate whether quetiapine could reduce sedative use in mechanically ventilated adults without delirium. Despite the addition of quetiapine, there was no significant reduction in 24 h doses of propofol, dexmedetomidine, or benzodiazepines. Similarly, in a recent retrospective study, the use of quetiapine was not advantageous over the other drugs in terms of efficacy and safety outcomes [10]. Different results were observed by Assadoon et al. [50], who found that atypical antipsychotics therapy—in this study quetiapine and olanzapine—was associated with a significant reduction in the dose of sedatives and analgesics administered. In a randomized double-blinded pilot study, treatment with quetiapine did not improve the number of days alive without delirium or coma, nor did it increase adverse outcomes [51].

Thus, the reason why the latest guidelines—although not specifically focused on the use of quetiapine for the treatment of delirium—have not provided strong recommendations regarding the use of antipsychotics is mainly due to the lack of a clinically meaningful effect on the duration of invasive mechanical ventilation [21].

In terms of the use of quetiapine for managing hyperactive delirium, few studies are available. A dated retrospective study compared the time to resolution of individual delirium symptoms in critically ill patients treated with quetiapine or with haloperidol rescue as placebo [52]. The results showed that quetiapine may lead to a faster resolution of symptoms like fluctuation, inattention, and disorientation, but a slower resolution of agitation and hyperactivity. A retrospective analysis comparing twice-daily vs bedtime dosing found no significant differences in delirium recovery or secondary outcomes (including ICU and hospital length of stay, mechanical ventilation duration, or in-hospital death) [53]. Both dosing schedules showed similar efficacy. Zahkary et al. [18] compared quetiapine to haloperidol for treating hyperactive delirium in 100 critically ill patients. The authors showed how the quetiapine group had a shorter ICU stay and more sleep hours per night, suggesting it may be as effective as haloperidol without affecting mortality.

The extent to which antipsychotics, including quetiapine, may reduce mortality remains questionable and is another key reason why updated guidelines have raised concerns about their use [21]. A case series demonstrated the potential use of quetiapine in managing refractory hyperactive and mixed ICU delirium [54]. The study involved 17 ICU patients who developed delirium after a median of five days. Quetiapine started at 25 mg daily, titrated to 50 mg, and resulted in a median delirium resolution time of four days. Patients showed a reduced need for other delirium medications. Adverse effects included somnolence and transient hypotension. Abraham et al. [53] hypothesized the use of quetiapine as a prophylactic treatment to prevent delirium in high-risk ICU patients. Their study involved 71 patients at high risk for delirium, with half receiving quetiapine (12.5 mg every 12 h) and the other half receiving no pharmacologic prophylaxis. The results showed a lower incidence of delirium in the quetiapine group (45.5% vs. 77.6%) and a significant reduction in ventilator duration. Regarding the treatment of hypoactive delirium, a retrospective study evaluated quetiapine among 113 adults, in which those treated with quetiapine had a shorter median delirium duration compared to untreated patients [55].

Several studies have shown that quetiapine is effective in reducing the duration and severity of delirium in ICU patients. Devlin et al. [56] conducted a double-blind, placebo-controlled trial demonstrating that quetiapine significantly shortened the duration of delirium compared to placebo, although these results were obtained on a low number of patients and have not been replicated. Similar findings were reported by Girard et al. [51], who observed a faster resolution of delirium symptoms with quetiapine compared to placebo.

The impact of quetiapine on ICU length of stay remains inconsistent [10]. Some studies suggest a reduction in ICU stay with quetiapine use attributed to the quicker resolution of delirium [56]. However, others have found no significant difference in ICU or hospital length of stay when compared to placebo or other antipsychotics [57].

**Table 1:** Principal investigations on quetiapine in critical care setting.

Study	Patients	Design	Main Findings
Martinez et al. [1]	665 ICU patients	Retrospective observational study	The screening rates for RASS and CAM-ICU were below the recommended levels. The administration of antipsychotic medications—mostly quetiapine in this cohort—occurs more frequently than the diagnosis of delirium
Sessler et al. [2]	192 ICU patients	Comparative study	RASS showed high inter-rater reliability among the entire adult ICU population. Robust correlations between the investigator-assigned RASS and the visual analog scale scores validated the use of RASS across all subgroups within the ICU. The RASS scores documented by individual physicians, nurses, and pharmacists exhibited a strong correlation with the principal investigator’s visual analog scale score.
Ely et al. [3]	290 ICU patients	Prospective cohort study	The RASS represents the first sedation scale validated for its capacity to identify variations in sedation levels over successive days of ICU treatment in relation to constructs such as consciousness levels and delirium, and it showed a correlation with the dosages of sedative and analgesic medications administered. This study confirmed the reliability and validity of RASS for monitoring sedation status over time.
Miranda et al. [4]	2817 ICU	Cochrane review	This study evaluated CAM-ICU for diagnosing delirium in critical care settings. The test is primarily beneficial for ruling out delirium. However, it may fail to identify a subset of patients with newly developed delirium. Consequently, in scenarios where comprehensive detection of all delirium cases is essential, it may be advisable to either retest or to use the CAM-ICU in conjunction with an additional assessment.
Marshall et al. [5]	164,996 ICU patients	Retrospective observational cohort study	Antipsychotic medications are prescribed to 1 in every 10 patients in the ICU, and their use is correlated with prolonged lengths of stay in both the ICU and the hospital. Patients receiving antipsychotics without any recorded diagnosis of a mental disorder exhibit longer ICU stays, extended hospitalizations, and higher mortality rates in comparison to those with a documented mental disorder.
Ohman et al. [49]	57 ICU mechanically ventilated	Retrospective interpatient comparator study	Following quetiapine initiation, no significant differences were observed in 24 h cumulative doses of propofol, dexmedetomidine, benzodiazepines, or opioids. However, dexmedetomidine requirements increased significantly over 48 h (*p* = 0.03). There were no changes in pain or sedation scores, sedation depth, or QTc interval.
Assadoon et al. [50]	107 ICU patients	Retrospective	Within 48 h of initiating atypical antipsychotics, 77.6% of patients showed a ≥20% reduction in cumulative dose of a sedative/analgesic. Propofol use decreased significantly, and dexmedetomidine use increased. Sedation levels were lighter, with no change in pain scores.
Wan et al. [54]	70 ICU patients	retrospective	In patients previously treated with multiple agents for delirium (median RASS = 3), quetiapine was started at 25 mg/day and titrated to 50 mg/day. Initiated after a median of 15 days of persistent delirium, quetiapine was associated with a reduced use of other medications and delirium resolution within a median of 4 days. Reported adverse effects included somnolence and transient hypotension.
Michaud et al. [55]	113 ICU diagnosed hypoactive delirium	Prospective interventional study	Quetiapine was associated with a shorter median duration of delirium (1.5 vs. 2.0 days, *p* = 0.04) and a trend toward faster extubation (3 vs. 5 days, *p* = 0.08). No significant differences were observed in ICU or hospital length of stay, and safety profiles were comparable between groups
Girard et al. [51]	101 ICU patients mechanically ventilated	Randomized, double-blind, placebo-controlled	Neither haloperidol nor ziprasidone significantly shortened the duration of delirium when compared to placebo. Patients across the three treatment groups had a comparable number of days alive without experiencing delirium or coma.
Mart et al. [58]	566 ICU patients	Randomized, double-blind, placebo-controlled	In critically ill patients experiencing delirium, neither haloperidol nor ziprasidone demonstrated a significant impact on cognitive, functional, psychological, or quality-of-life outcomes in survivors.
Devlin et al. [56]	36 ICU patients with delirium	Prospective, randomized, double-blind, placebo-controlled	The inclusion of quetiapine with as-needed haloperidol is associated with a more rapid resolution of delirium, decreased levels of agitation, and a higher rate of discharge to home or rehabilitation. Patients receiving quetiapine need fewer days of as-needed haloperidol. Furthermore, the occurrence of QTc prolongation and extrapyramidal symptoms was comparable between the groups.
Zakhary et al. [18]	100 ICU patients	Randomized controlled	Quetiapine has been shown to be as effective as haloperidol in alleviating the symptoms of hyperactive delirium in critically ill patients, although it does not confer any benefit in terms of mortality.
Maneeton et al. [57]	52 ICU patients with delirium	Prospective, double-blind, randomized controlled	Low doses of quetiapine and haloperidol demonstrate comparable efficacy and safety for managing behavioral disturbances (efficacy, tolerability, total sleep time) in patients with delirium, particularly when combined with environmental modifications.
Alghadeer et al. [10]	47 ICU patients	Retrospective comparative study	The authors found no significant differences in efficacy or adverse effects when comparing the treatment of delirium with quetiapine, haloperidol, risperidone, and olanzapine.
Wang et al. [11]	11,173 patients, quetiapine vs. haloperidol	Multi-center retrospective cohort study	The authors showed that severe QT prolongation was prevalent among patients undergoing treatment with quetiapine or haloperidol. A considerable proportion of these patients were exposed to risk factors associated with QT prolongation, including older age, heart failure, hypokalemia, and the concurrent administration of medications recognized to elevate the risk of torsades des pointes.
Dube et al. [59]	103 ICU patients	Single-center, prospective cohort analysis	There were no reported occurrences of torsades de pointes. QTc prolongation was relatively rare among critically ill patients receiving quetiapine. Patients who were prescribed concomitant medications known to prolong the QTc interval may be at a heightened risk.
Tomichek et al. [60]	500 ICU patients	Single-center prospective cohort study	The administration of an atypical antipsychotic markedly increased the probability of receiving an antipsychotic prescription at discharge, a practice that should be carefully evaluated during medication reconciliation

## 6. Caution and Contraindications

Quetiapine is generally well-tolerated by patients, but its safety profile requires the careful consideration of several key aspects. One notable concern is its potential to prolong the QTc interval [11]. While quetiapine can cause a slight prolongation of this interval, the actual risk of inducing a serious arrhythmia known as torsade de pointes is very low. A comprehensive review identified only four cases of torsade de pointes associated with quetiapine, all of which involved additional significant risk factors [61]. Thus, although caution is necessary for patients with pre-existing QT prolongation, quetiapine alone poses minimal risk in this regard [59].

Another important aspect of quetiapine’s safety profile is its low incidence of extrapyramidal side effects [62]. The studies by Mistraletti et al. [63] and Tomichek et al. [60] report low incidences of extrapyramidal symptoms and no significant differences in mortality rates between quetiapine and placebo groups.

Quetiapine has the potential to cause hypotension [64]. However, this effect has not been specifically studied in ICU settings. Nonetheless, the constant monitoring typical of ICU care can facilitate its early detection, although hypotension in critically ill patients is often multifactorial. Hepatic dysfunction can affect the drug’s processing, potentially necessitating dose adjustments to avoid adverse effects and ensure optimal efficacy. Consequently, the close monitoring of liver function is essential, with adjustments made as necessary [65]. Conversely, renal dysfunction does not typically require changes in quetiapine dosing, as the drug’s elimination is not significantly impacted by kidney function [47]. A report presented results from two small studies investigating the pharmacokinetics of quetiapine in nonpsychotic subjects with renal impairment [66]. Equal numbers of impaired and healthy control subjects received a single 25 mg dose, with plasma concentrations measured for up to 48 h.

## 7. Clinical Practical Algorithm

**Figure 1:**
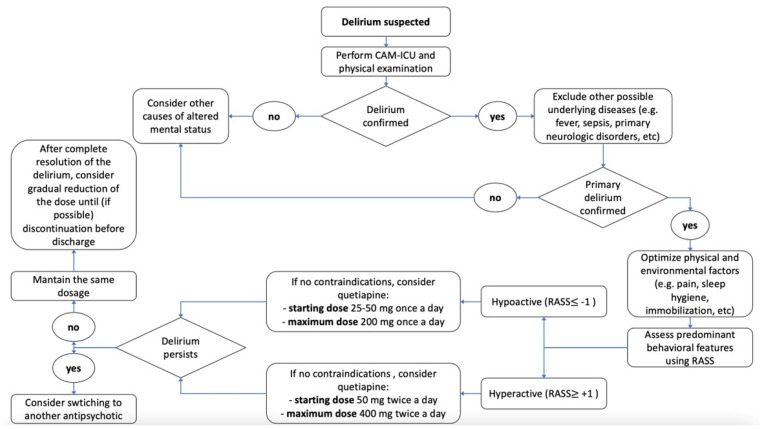
Practical approach to managing delirium in the ICU.

Based on the observations described above, balancing the broader guideline recommendations with the specific data we have gathered on quetiapine, we proposed a practical approach for managing patients with delirium in the ICU, as illustrated in Figure 1. The figure is not intended as a formal guideline but rather as a practical suggestion to help clinicians navigate the complexities of managing ICU delirium with quetiapine. The initial step in evaluating a patient exhibiting the characteristic clinical signs of delirium is to conduct the CAM-ICU assessment [4]. If delirium is confirmed, it is essential to identify and rule out all potential underlying factors or conditions that may contribute to its onset, including fever, sepsis, and primary neurological disorders. Similarly, it is crucial to optimize environmental factors. This includes implementing effective pain management, minimizing noise and light to promote good sleep hygiene, and initiating mobilization as soon as feasible [67]. After optimizing all these factors, it is advisable to assess the level of sedation and agitation using the RASS, allowing for the differentiation of delirium based on behavioral characteristics into hypoactive and hyperactive subtypes [68].

If the patient has no contraindications (such as prolonged QTc interval, or concurrent use of contraindicated medications like azole antifungals, erythromycin, or clarithromycin), the initiation of treatment with quetiapine may be considered. For a patient with hypoactive delirium, treatment should be initiated with 25–50 mg once daily [55,69], with the possibility of increasing the dose to a maximum of 200 mg once daily [10].

For a patient with hyperactive delirium, the starting dose should be 50 mg twice daily, which can be escalated to a maximum of 400 mg twice daily [18,36,54]. If the treatment is ineffective, a change in antipsychotic should be considered.

Conversely, if the therapy is effective, it is advisable to maintain the dosage until complete resolution is achieved. Once the delirium has fully resolved, it is crucial to consider a gradual reduction in the dosage and, if feasible, the discontinuation of the medication prior to discharge [68].

This figure, created by the authors, outlines a step-by-step process for managing ICU patients with delirium, based on concepts derived from references [4,10,18,36,58,59,62,68,69]. It begins with the CAM-ICU assessment to confirm diagnosis. Once delirium is identified, it is essential to search for and address any underlying causes, such as infections or neurological issues, while also optimizing the patient’s environment (pain control, promoting sleep, and encouraging early mobilization). After these factors are optimized, the patient’s sedation and agitation levels should be assessed using the RASS, helping to distinguish between hypoactive and hyperactive delirium. If no contraindications are present, treatment with quetiapine can be considered, with tailored dosing based on the delirium subtype. Treatment should be regularly reviewed and adjusted based on clinical response, and once delirium resolves, a gradual dose reduction is recommended, ideally aiming for discontinuation before discharge.

## 8. Conclusions

Quetiapine seems to be a reasonable option for managing delirium in ICU patients, showing promising results in reducing both delirium duration and agitation. Its safety profile appears acceptable overall, with side effects that are generally manageable. However, its effect on ICU length of stay remains uncertain, highlighting the need for further investigation. The current evidence is limited by the wide variability in study designs, patient populations, and outcome measures, making it difficult to draw firm conclusions. While available evidences are encouraging, larger, well-conducted trials are essential to better understand both its short-term effectiveness and its long-term impact.
